# Efficacy and safety of platelet-rich plasma for the treatment of thin endometrium: A protocol for systematic review and meta-analysis

**DOI:** 10.1097/MD.0000000000018848

**Published:** 2020-01-17

**Authors:** Juan Du, Hua Lu, Xujun Yu, Zili Lü, Ling Mi, Xiaojin Zhang

**Affiliations:** a Hospital of Chengdu University of Traditional Chinese Medicine; b Chengdu University of Traditional Chinese Medicine; c The Reproductive and Women-Children Hospital, Chengdu University of Traditional Chinese Medicine, Chengdu, Sichuan, China.

**Keywords:** platelet-rich plasma, thin endometrium, meta-analysis, systematic review, protocol

## Abstract

**Background:**

Endometrial thickness has been identified as a prognostic factor for pregnancy rate for patients with female infertility. Thin endometrium is defined as <7 mm on the day of ovulation, or on the day of human chorionic gonadotrophin (HCG) injection in fresh in vitro fertilization (IVF) cycles, or the day to start progesterone in frozen-thaw embryo transfer cycles, as stated in the guideline of thin endometrium from the Canadin Fertility and Andrology Society and Chinese expert consensus from the Society of Reproductive Medicine, Chinese Medical Association. Many medicines are used for improving the endometrial thickness and embryo implantation rate of the patients with thin endometirum, but thin endometrium remains a major troublesome clinical problem with limited efficacy.

Platelet-rich plasma (PRP), as a growing and robust therapeutic option in musculoskeletal medicine, is a preparation of autologous plasma with a high concentration of platelets, and the therapeutic mechanism is based on the capacity to supply supra physiologic amounts of essential growth factors to provide a regenerative stimulus for promoting repair in tissues with low healing potential. Some randomized controlled trials have reported the application of PRP for patients with thin endometrium with satisfactory effect. However, there is no systematic review on efficacy and safety of PRP as a treatment of thin endometrium.

**Methods:**

The data and information will be retrieved from the databases of MEDLINE, Embase, Web of Science, Clinicaltrials.org., Cochrane Library, China Biology Medicine Database, Wan Fang Database, China National Knowledge Infrastructure Database, VIP Science Technology Periodical Database, and OpenGrey for gray literature. The randomized controlled clinical trials are going to be selected before December 20, 2019, in English or Chinese language, with the search terms including “thin endometrium,”“platelet-rich plasma,” “endometrial thickness,” “hemorheology of endometrium,” “pregnancy rate,” and “adverse reactions.” RevMan 5.3 will be used for systematic review and meta-analysis. This protocol will be reported according to the Preferred Reporting Items for Systematic Reviews and Meta-Analyses Protocols (PRISMA-P) statement, and the systematic review will be reported with the PRISMA statement.

**Results and conclusion:**

The efficacy and safety of PRP for the treatment of thin endometrium will be evaluated, and the conclusion will be published to provide medical evidence for a better clinical decision of patients with thin endometirum.

## 1 Introduction

Infertility is a disease characterized by the failure of clinical pregnancy after 12 months regular sexual intercourse, without conception. It is estimated that the incidence rate of infertility is between 8% and 12% in reproductive-aged couples worldwide.^[1]^ For female infertility of the factors that prevent normal implantation and pregnancy, embryo and endometrial quality share responsibility.^[2]^

Thin endometrium is defined as <7 mm on the day of ovulation, or on the day of human chorionic gonadotrophin (HCG) injection in fresh in vitro fertilization (IVF) cycles, or the day to start progesterone in frozen-thaw embryo transfer cycles.^[3,4]^

A large number of studies had already fully proved that endometrial thickness and pattern were independent factors which affect pregnancy outcomes, and thin endometrium was considered as an independent negative prognostic factor for achieving pregnancy in women, whether or not with ovarian stimulation.^[5–8]^ And the endometrial thickness even had an influence on pregnant women or children.^[9,10]^ The incidence rate of thin endometrium is about 2.4% in the assisted reproductive technology cycles, and is associated with lower implantation rate and pregnancy rate.^[11]^ Treatment of “thin endometrium” remains a challenge, and large researches are required to further elucidate and optimal management of patients with “thin” endometrium in the future.^[12]^ Platelet-rich plasma (PRP) with a high concentration of platelets is able to supply supra physiologic amounts of essential growth factors to provide a regenerative stimulus for promoting repair in tissues with low healing potential.^[13]^ Some randomized controlled trials had reported the application of PRP for patients with thin endometrium, and found that it was effective to improve the endometrial growth and pregnancy outcomes.^[14–16]^ However, there is no systematic review on efficacy and safety of PRP for the treatment of thin endometrium, so our reviewer teams begin to do this job.

### 1.1 Review objectives

This review is aimed to evaluate the efficacy and safety of PRP for the treatment of patients with thin endometrium, comparing with another treatment, placebo or no treatment. The main out measures are endometrial thickness, hemorheology of endometrium, pregnancy rate, and adverse reactions. The conclusion will be published to provide medical evidence for better clinical decision to treat patients with thin endometirum.

## 2 Methods

This is a systematic review, with meta-analysis if necessary. Almost all the data and information are extracted from the published articles and studies, so it does not require ethical approval.

### 2.1 Protocol and registration

This review is registered on PROSPERO with the registration number: PROSPERO CRD42019148924.

This protocol will be reported according to the Preferred Reporting Items for Systematic Reviews and Meta-Analyses Protocols (PRISMA-P) statement, and the systematic review will be reported with the PRISMA statement.^[17]^

### 2.2 Data source

#### 2.2.1 Electronic search database and approach

The data and information will be retrieved from the databases of MEDLINE, Embase, Web of Science, Clinicaltrials.org., Cochrane Library, China Biology Medicine Database (CBM), Wan Fang Database, China National Knowledge Infrastructure Database (CNKI), and VIP Science Technology Periodical Database. The randomized controlled clinical trials (RCTs) are going to be selected before December 20, 2019, in English or Chinese language, with the search terms including “thin endometrium,” “platelet rich plasma,” “endometrial thickness,” “hemorheology of endometrium,” “pregnancy rate,” “adverse reactions,” and the Chinese key words corresponding to the words above. Use the right retrieval strategy for different databases, and Table 1 shows the search example for MEDLINE.

**Table 1:**
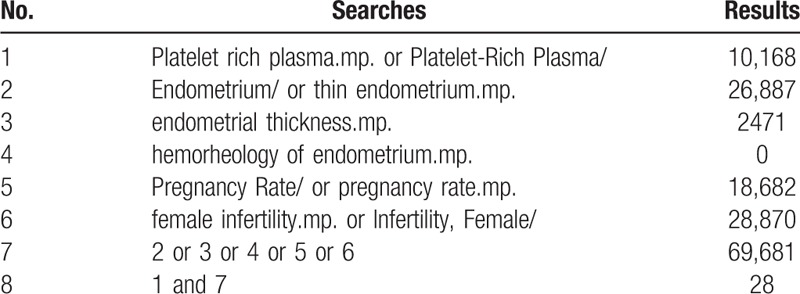
MEDLINE search strategies.

#### 2.2.2 Other sources of search

The data and information of gray literature will be retrieved from OpenGrey. Full texts will be obtained through electronic library or Internet. Conference papers can be obtained by contacting the author for the original research detail. Manual retrieval of references will assist to identify studies related to this review.

### 2.3 Included and excluded criteria

#### 2.3.1 Study design

Only human RCT studies can be included in the review, while other kinds of study will be excluded, such as observational studies, retrospective analyses, self-controlled trials, case reports, reviews, patient series, animal experiments, etc.

#### 2.3.2 Participants

##### 2.3.2.1 Included population

Female infertility patients of childbearing age who have fertility requirements and the maximal endometrial thickness by B ultrasound scanning was below 7 mm on the day of ovulation, or on the day of HCG administration in fresh IVF cycles, or the day of start of progesterone in frozen-thaw embryo transfer cycles.^[3,4]^

##### 2.3.2.2 Excluded population

Patients suffered from uterine or endometrial dysplasia or structural abnormalities except for thin endometrium.

#### 2.3.3 Interventions

Treatment group consists of the patients with thin endometrium treated with PRP.

Control group: Placebo-controlled group consists of the patients with a placebo in the same appearance, or another treatment group with one of estrogen, gonadotropins, letrozole, granulocyte colony-stimulating factor, sildenafil citrate, pentoxifyllin, tocopherol, and tamoxifen, or no treatment group.

#### 2.3.4 Outcomes

##### 2.3.4.1 Primary outcome indicator

1. Endometrial thickness: Endometrial thickness was measured as the maximum distance between the 2 interfaces of the endometrial–myometrial junction, in the midsagittal plane of the uterus by B ultrasound radiography.^[6]^
2. Clinical pregnancy rate: Clinical pregnancy was defined as the identification of a gestational sac with fetal heart activity on B ultrasound examination 4 to 5 weeks after embryo transfer and clinical pregnancy rate is expressed per cycle.^[6]^
3. Adverse reactions.

It will be based on the results reported at the end of included studies.

##### 2.3.4.2 Secondary outcome indicators

1. Miscarriage rate: Miscarriage was defined as spontaneous pregnancy loss after sonographic visualization of an intrauterine gestational sac. Miscarriage rate is expressed per clinical pregnancy cycle.
2. Endometrial pattern: Endometrial pattern by B ultrasound radiography were classified according to the morphology of the endometrium as: pattern A (triple-line type which is characterized by a hypoechoic endometrium with well-defined hyper-echoic outer walls and a central echogenic line); pattern B (isoechoic endometrium with poorly defined outer walls and central echogenic line); pattern C (homogeneous hyper-echoic endometrium).^[18]^
3. Live birth rate: Live birth rate was classified as those cycles resulting in the delivery of a live infant after 24 weeks gestation. Live birth rate is expressed per treatment cycle.

### 2.4 Selection of studies and data extraction

Two well-trained reviewers (JD, XZ) make retrieval strategies, respectively, and manage all the documents by the software Endnote X9. Firstly, filter and delete the duplicate studies from the information like titles and abstracts, and then scan all the articles which comparing PRP with placebo-controlled group, or other treatment group, or no treatment group according to the inclusion criteria and exclusion criteria. The appropriate articles need to be further studied and searched for full-text data.

**Figure 1:**
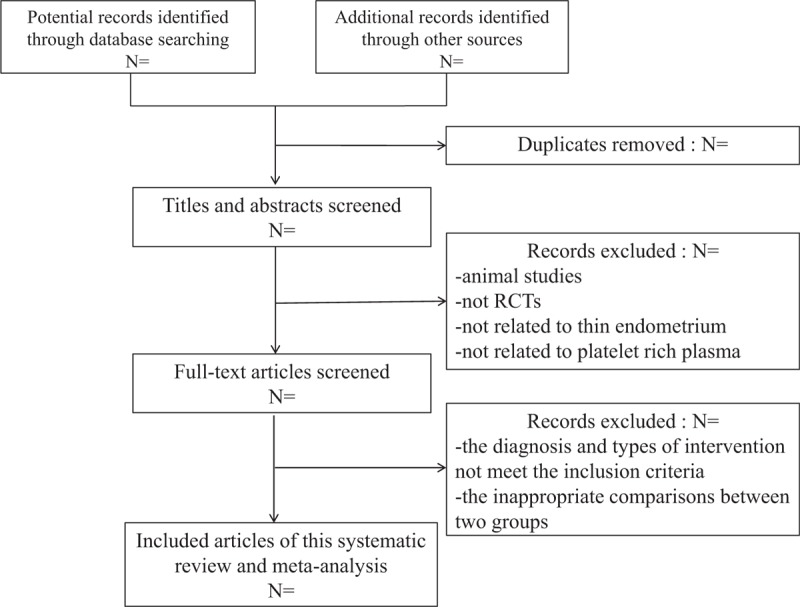
The PRISMA literature screening flow chart.

Two review authors will extra data and information separately and simultaneously, including characteristics and methodology, participant characteristics, details of interventions and so on (Fig. 1). Before the extraction of formal data, all controversial issues in the process will be discussed and resolved with the third professional reviewer (XY). When there are some questions about the original data or some missing information of conference studies, the review team will contact the corresponding author by email or phone.

### 2.5 Risk of bias assessment

The biases consist of selection bias, performance bias, detection bias, attrition bias, and reporting bias according to the Cochrane Collaboration Network Risk Assessment Tool. Each of the 2 well-trained reviewers will evaluate the risk of bias by himself. If it cannot be determined, a discussion will be carried out with the 3rd review author.

And then the risk of bias assessment chart of inclusion studies will be drawn up with the software RevMan 5.3.

### 2.6 Data analysis and synthesis

When the data of different studies can be combined or the studies are homogeneous without heterogeneity, meta-analysis can be conducted by software RevMan 5.3, otherwise the descriptive analysis is necessary. The dichotomous variable should be merged to risk ratio (RR) and 95% confidence intervals, while the continuous variable will be merged to mean difference (MD) and 95% confidence intervals. And Cochran *Q* statistic and *I*^2^ statistic are used for calculating the indicators reflecting heterogeneity, such as *P* < .10 means heterogeneous, and *I*^2^ > 50% is significant heterogeneity. When *I*^2^ < 50%, a fixed effect model (Mantel–Haenzel method for RR and inverse variance for MD) is chosen, as well as when the heterogeneity is still significant after sensitivity analysis and subgroup analysis, a random effects model (D-L method) is suitable. It is considered to be statistically significant with *P* < .05 in *Z* test.

### 2.7 Subgroup analysis

If there is obvious heterogeneity between the included studies, and the heterogeneity will be significantly reduced by dividing into subgroup of different ages, or different sorts of disease which leads to thin endometrium, or appropriate interventions, the subgroup analysis will be performed.

### 2.8 Sensitivity analysis

Sensitivity analysis is conducted to measure the stability and reliability of the meta-analysis result, and search for the causation of heterogeneity. Some factors as the methodologic quality and sample size are the critical points. Getting rid of the studies with high risk of bias or removing some special studies is a solution for sensitivity analysis.^[18]^

### 2.9 Publication bias

Publication bias is commonly associated with inflated treatment effect which lowers the certainty of decision makers about the evidence. Various statistical approaches are available to determine the direction and magnitude of publication bias.^[19,20]^ Published bias will be evaluated by a funnel plot with the software Review Manager 5.3, Begg test, and Egger test by Stata software 14.0.^[20]^

## 3 Discussion

Human endometrium has a key role in embryo implantation process. The measurement of endometrial thickness is the most commonly used in clinical practice. Managing patients with thin endometrium still represents a major challenge for clinicians.^[21]^

A variety of drugs currently are used to improve thin endometrium, such as estrogen, gonadotropins, letrozole, granulocyte colony-stimulating factor, sildenafil citrate, pentoxifyllin, tocopherol, tamoxifen, etc. The PRP is also used to treat this disease, and it is listed in the management of thin endometrium in assisted reproduction of the clinical guideline published in July 2019 from the Canadian Fertility and Andrology Society, but the level of evidence recommended is just weak.^[1]^ After the publishment of the guideline, there are still some RCT studies on it to prove its effectiveness. So systematic review on the efficacy and safety of PRP for the treatment of thin endometrium is necessary.

The limitations of this systematic review show the reviewer authors focus on the articles and researches in English and Chinese inside of other languages, and there is still small amount of the RCTs now and we are waiting for more studies on it with hope, because although it has been widely used in the field of Orthopedics and Dermatology, PRP is still a new application in Gynecology and reproductive medicine for treating thin endometrium.

## Author contributions

**Data management:** Juan Du, Xujun Yu, Hua Lu

**Draft writing:** Juan Du, Xujun Yu, Xiaojin Zhang, Ling Mi

**Manuscript modification and editing:** Juan Du, Xiaojin Zhang, Zili Lü

**Methodology:** Juan Du, Xujun Yu, Hua Lu, Ling Mi

**Program management:** Juan Du, Xiaojin Zhang, Zili Lü, Hua Lu

**Research design and concept:** Juan Du, Hua Lu, Xiaojin Zhang

**Resource:** Hua Lu, Xujun Yu

**Review the manuscript and approve the release:** Juan Du, Xiaojin Zhang, Ling Mi

**Software:** Xiaojin Zhang, Juan Du, Zili Lü
